# Longitudinal Circulating Levels of miR-23b-3p, miR-126-3p and lncRNA GAS5 in HCC Patients Treated with Sorafenib

**DOI:** 10.3390/biomedicines9070813

**Published:** 2021-07-13

**Authors:** Michele Manganelli, Ilaria Grossi, Manuela Ferracin, Paola Guerriero, Massimo Negrini, Michele Ghidini, Chiara Senti, Margherita Ratti, Claudio Pizzo, Rodolfo Passalacqua, Sarah Molfino, Gianluca Baiocchi, Nazario Portolani, Eleonora Marchina, Giuseppina De Petro, Alessandro Salvi

**Affiliations:** 1 Department of Molecular and Translational Medicine, Division of Biology and Genetics, University of Brescia, 25123 Brescia, Italy; m.manganelli@unibs.it (M.M.); ilaria.grossi@unibs.it (I.G.); eleonora.marchina@unibs.it (E.M.); 2 Department of Experimental, Diagnostic and Specialty Medicine, University of Bologna, 40126 Bologna, Italy; manuela.ferracin@unibo.it; 3 Department of Morphology, Surgery and Experimental Medicine, University of Ferrara, 44121 Ferrara, Italy; grrpla@unife.it (P.G.); massimo.negrini@unife.it (M.N.); 4 Department of Oncology, Azienda Socio Sanitaria Territoriale of Cremona, 26100 Cremona, Italy; m.ghidini@asst-cremona.it (M.G.); chiara.senti@gmail.com (C.S.); m.ratti@asst-cremona.it (M.R.); claupizz1974@gmail.com (C.P.); r.passalacqua@asst-cremona.it (R.P.); 5 Department of Clinical and Experimental Sciences, Surgical Clinic, University of Brescia, 25123 Brescia, Italy; sarahmolfino@gmail.com (S.M.); gianluca.baiocchi@unibs.it (G.B.); nazario.portolani@gmail.com (N.P.)

**Keywords:** HCC, sorafenib, ddPCR, GAS5, miR-126-3p, miR-23b-3p

## Abstract

Human hepatocellular carcinoma (HCC) is the most frequent primary tumor of the liver and the third cause of cancer-related deaths. The multikinase inhibitor sorafenib is a systemic drug for unresectable HCC. The identification of molecular biomarkers for the early diagnosis of HCC and responsiveness to treatment are needed. In this work, we performed an exploratory study to investigate the longitudinal levels of cell-free long ncRNA GAS5 and microRNAs miR-126-3p and -23b-3p in a cohort of 7 patients during the period of treatment with sorafenib. We used qPCR to measure the amounts of GAS5 and miR-126-3p and droplet digital PCR (ddPCR) to measure the levels of miR-23b-3p. Patients treated with sorafenib displayed variable levels of GAS5, miR-126-3p and miR-23b-3p at different time-points of follow-up. miR-23b-3p was further measured by ddPCR in 37 healthy individuals and 25 untreated HCC patients. The amount of miR-23b-3p in the plasma of untreated HCC patients was significantly downregulated if compared to healthy individuals. The ROC curve analysis underlined its diagnostic relevance. In conclusion, our results highlight a potential clinical significance of circulating miR-23b-3p and an exploratory observation on the longitudinal plasmatic levels of GAS5, miR-126-3p and miR-23b-3p during sorafenib treatment.

## 1. Introduction

Non-coding RNAs (ncRNAs) can be classified into two main classes according to their size, namely small non-coding RNAs (sncRNAs) if shorter than 200 nt or long non-coding RNAs (lncRNAs) if longer than 200 nt [1]. Among the sncRNAs, microRNAs (miRs) are the most studied and characterized class. ncRNAs play a distinct role in both physiological and pathological processes [2,3,4,5]; they also reside in the different anatomical and biological districts [6] and show cell/tissue-specific expression patterns as seen in different types of tumors [7,8].

Hepatocellular carcinoma (HCC) is the most common primary tumor of the liver and the third cancer-related cause of death worldwide [9]. It is a heterogeneous malignant tumor, often diagnosed at an advanced stage and therefore with a poor prognosis. FDA (Food and Drug Administration) and EMA (European Medicines Agency) approved the drug sorafenib (sf) and lenvatinib as first-line treatments for HCC in advanced unresectable stage and not eligible for local treatments. Sorafenib and lenvatinib proved to significantly extend the median survival time of HCC patients, with lenvatinib non-inferior to sorafenib. However, they can cause adverse side effects, and some patients develop secondary resistance during treatment [10,11]. Recently, atezolizumab with bevacizumab combined immunotherapy resulted in better overall and progression-free survival outcomes than sorafenib in unresectable HCC [12]. However, nowadays, immunotherapy is not globally accessible; therefore, the use of sorafenib is still taken into account. Sorafenib acts as a serine/threonine and a tyrosine kinases inhibitor in multiple oncogenic signalling pathways (CRAF, BRAF, eIF4E, VEGFR-2 and -3, PDGFR-β, FGFR-1, c-kit, FLT-3 and RET) in both the tumor and the endothelial cells or pericytes [13]. Among sorafenib-induced effects, there are variations in mRNA transcripts, proteome, DNA methylome as well as dysregulation of ncRNAs [14,15,16,17,18].

The EASL (European Association for the Study of the Liver) guidelines identified the development of new tools with high sensitivity in predicting and monitoring therapeutic response or resistance to systemic therapy as an unmet need in HCC research [19]. In this context, it is a common opinion that the use of circulating ncRNAs as biomarkers may be considered useful if a reliable and sensitive method could be available.

In this work, we directed our attention to plasmatic circulating levels of the lncRNA GAS5, miR-126-3p and miR-23b-3p [20,21,22,23]. GAS5 (Growth Arrest Specific 5) is involved in cell cycle regulation, proliferation, apoptosis, cell migration and invasion [24]. It is downregulated in a variety of malignancies, including HCC [25,26]. We previously reported that the expression of GAS5 was significantly upregulated in the HCC cell line HA22T/VGH when the cells were treated with sf compared to untreated ones [20]. miR-126-3p is involved in cell proliferation, migration, angiogenesis, vascular integrity and inflammation, and therefore, it is associated with microvascular invasion, tumor early recurrence and metastasis [27,28,29]. In HA22T/VGH cells, we demonstrated that the expression of miR-126-3p was downregulated when treated with sf compared to untreated cells [20]. We also previously reported that GAS5 and miR-126-3p were found respectively in lower and higher amounts in the plasma of untreated HCC patients compared to healthy individuals, potentially serving as diagnostic molecules. Finally, miR-23b-3p is involved in cell proliferation, migration and invasion. It has been extensively studied in different types of human cancer [21], and its dysregulation has been found in a variety of malignancies. In particular, we demonstrated that the expression of miR-23b-3p was significantly downregulated in HCC tissues compared to their peritumoral counterparts [22,23]. To our knowledge, no data are available concerning miR-23b-3p as a circulating molecule in plasma of HCC patients: for this reason, in the present work, we measured by ddPCR the plasma levels of miR-23b-3p in a cohort of 25 unresectable HCC patients and we compared them with those of 37 healthy individuals. Finally, no data are available about GAS5, miR-126-3p and miR-23b-3p levels in HCC patients during the treatment with sorafenib. Here, we present an exploratory work to determine the levels of GAS5, miR-126-3p and miR-23b-3p levels in plasma from liquid biopsies of HCC patients at different time points of follow-up.

## 2. Materials and Methods

### 2.1. Liquid Biopsies Collection of Sorafenib Treated HCC Patients

Peripheral blood samples were collected in EDTA-coated collection tubes. The peripheral blood (10 mL/patient) of 7 HCC patients (Table S1) treated with sorafenib was collected before the starting of the treatment (T_0_) and at 1-month intervals after the treatment started at the Oncology Division of ASST Cremona Hospital (Italy). Plasma was obtained by centrifugation of peripheral blood at 3000 rpm for 10 min at 4 °C. All recruited patients were screened for virus-related disease and background pathology. All patients received 800 mg/day of sorafenib (400 mg twice/day) as an initial dosage. Dosage reduction occurred with the appearance of toxicity or adverse effects. Patients continued treatment unless there was a clinical progression, unacceptable toxicity, refusal to continue or death.

### 2.2. Liquid Biopsies Collection of Untreated HCC Patients and Healthy Individuals

Ten mL/person of peripheral blood were collected in VACUETTE^®^ blood collection tubes containing EDTA as anti-coagulant from 37 healthy volunteers (mean age = 46.78 y ± 7.6 SD) at the Immunohematology and Transfusion Medicine Service of ASST *Civili* Hospital in Brescia (Italy). Peripheral blood (10 mL/patient) was collected in VACUETTE^®^ blood collection tubes containing EDTA as anti-coagulant from 25 untreated HCC patients (Table S2) before HCC resection at the Surgical Clinic of ASST *Civili* Hospital in Brescia (Italy). Plasma was obtained from 1 mL of peripheral blood within 1 h from the withdrawal by spinning it at 3000 rpm for 10 min at 4 °C followed by a second centrifugation step at 4000 rpm for 20 min at 4 °C. Samples showing hemolysis were excluded [30,31]. The plasma was transferred to a new tube and stored at −80 °C until RNA extraction. The study was approved by both the ethical committees at ASST *Civili* Hospital of Brescia on 2 October 2012 (NP1230) and at ASST Cremona Hospital on 28 December 2015 (NP30769). Informed consent was obtained from all the subjects enrolled in the study. All methods were performed in accordance with the relevant guidelines and regulations.

### 2.3. RNA Isolation from Plasma

Total RNA was isolated from 200 µL of plasma using miRNeasy Mini Kit (Qiagen; cat. n. 217004), according to the manufacturer’s instructions. After samples were mixed with 1 mL of QIAzol Lysis Reagent, 2.5 μL of a 5 nmol/µL solution of the synthetic miRNA cel-miR-39-3p from *C. elegans* (Integrated DNA Technologies; Coralville, IA, USA) were added. RNA was eluted from spin columns in 35 μL nuclease-free water.

### 2.4. Quantitative Real-Time PCR (qPCR)

To measure the amount of circulating GAS5, 2.5 µL of total RNA from plasma were retrotranscribed and pre-amplified using iScript Explore One-Step RT and PreAmp kit (Bio-Rad, Hercules, CA, USA; cat. n. 12004856) according to manufacturer’s instructions. The cDNA was diluted at 1:10, and 1.5 µL was used for qPCR analysis (RPLP0 Assay ID: Hs.PT.39a.22214824, GAS5 Assay ID: Hs.PT.5824767969; IDT) using 2× TaqMan Universal Master Mix (Applied Biosystems, Carlsbad, CA, USA; cat. n. 4440040).

To measure the amounts of circulating miR-126-3p, cDNA was synthesized from 2.5 µL of total RNA from plasma using the TaqMan microRNA Reverse Transcription Kit components (ThermoFisher, Carlsbad, CA, USA; cat. n. 4366596) and the stem-loop primer for miR-126-3p (Applied Biosystems; Assay ID: 002228) and the spike-in cel-miR-39 (Applied Biosystems; Assay ID: 000200) according to the manufacturer’s instructions. Each reaction was performed in triplicate. Data analysis was performed by a 7500 Real-Time PCR System (Applied Biosystems). Gene expression was measured with the 2^−ΔΔCt^ method, and cel-miR-39 and RPLP0 were used as a reference for RQ (relative quantification) determination.

### 2.5. Droplet Digital PCR Workflow

For miR-23b-3p, 3 μL of purified RNA were reverse transcribed in a 20 μL reaction using the miRCURY Locked Nucleic Acid Universal Reverse Transcription (RT) miRNA PCR, Polyadenylation and cDNA synthesis kit (Exiqon Inc., Vedbaek, Denmark; cat. n. 339340). cDNA was diluted at 1:50, and PCR was performed according to the QX200 EvaGreen ddPCR protocol. In brief, 8 μL of diluted cDNA, 10 μL of 2× EvaGreen Supermix (Bio-Rad, Hercules, CA, USA cat. n. 1864034) and 1 μL of miRCURY LNA PCR primer sets (Exiqon, Woburn, MA, USA; cat. n. 339306) for miR-23b-3p were mixed in a final volume of 20 μL. Each ddPCR assay mixture (20 μL) was loaded into a disposable droplet generator cartridge (Bio-Rad; cat. n. 1864007). Then, 70 μL of droplet generation oil for probes (Bio-Rad; cat. n. 1863005) was loaded into each of the eight oil wells. The cartridge was then placed inside the QX200 droplet generator (Bio-Rad). When droplet generation was completed, the droplets were transferred to a 96-well PCR plate (Bio-Rad; cat. n. 12001925) using a multichannel pipette. The plate was heat-sealed with foil and placed in a conventional thermal cycler. Thermal cycling conditions were: 95 °C for 5 min, then 40 cycles of 95 °C for 30 s and 58 °C for 1 min (ramping rate reduced to 2%), and three final steps at 4 °C for 5 min, 90 °C for 5 min and a 4 °C indefinite hold. A no-template control (NTC) and a negative control for each reverse transcription reaction (RT-neg) were included in every assay. The Cel-miR-39 assay was performed to monitor RT reaction efficacy.

### 2.6. Statistical Analysis

Statistical analysis was carried out using GraphPad Prism 7.0 software (San Diego, CA, USA). Mann–Whitney U-test was used to analyze the amount of miR-23b-3p in the plasma of healthy individuals compared to one of the HCC patients. Diagnostic performance of circulating miR-23b-3p to distinguish HCC patients from healthy subjects was evaluated using Receiver Operating Characteristic (ROC) curve analysis. The associations between the clinicopathological characteristics of HCC patients and the plasmatic levels of miR-23b-3p were calculated using Fisher’s exact test. The median value of copies/µL was used as a cut-off to classify HCC patients into low- (copies/µL ≤ 305) and high-circulating miR-23b-3p level (copies/µL > 305) groups. One-way ANOVA followed by Tukey’s test was used to compare ncRNAs levels among T_0_, T_1_ and T_2_. A *p*-value ≤ 0.05 was considered statistically significant.

## 3. Results

### Amounts of GAS5, miR-126-3p and miR-23b-3p in the Plasma of Patients Treated with Sorafenib

**Figure 1:**
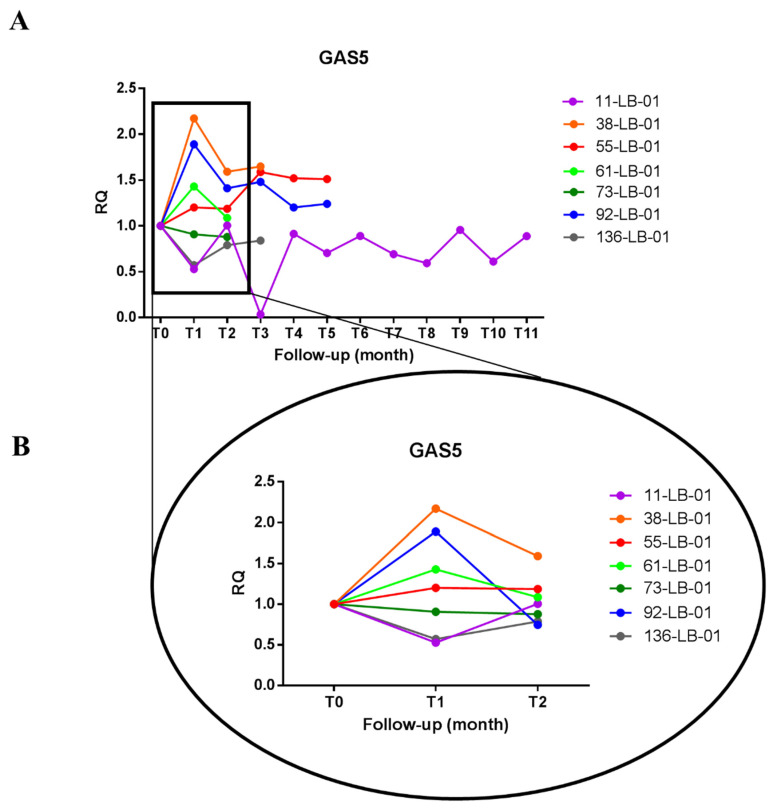
GAS5 levels in the plasma of HCC patients treated with sorafenib. (**A**) The graph shows the amount of GAS5 detected in the plasma of each HCC patient during sorafenib treatment. Each line represents one patient, and the dots indicate the levels of GAS5 at each blood withdrawal time point (one per month, Tn) normalized on the GAS5 level before the start of treatment (T0). (**B**) Focus on GAS5 early levels after sorafenib administration. T_n_ indicates the sequential blood withdrawal time point carried out once *per* month. One-way ANOVA, followed by Tukey’s test, was used to compare GAS5 levels among three time points (T0, T1 and T2).

**Figure 2:**
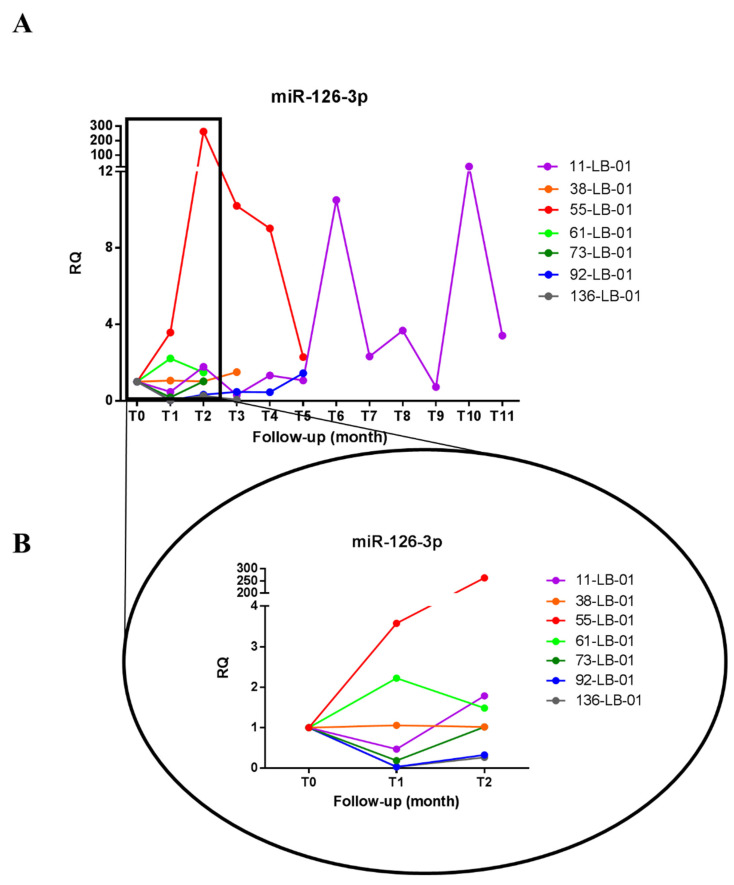
miR-126-3p levels in the plasma of HCC patients treated with sorafenib. (**A**) The graph shows the amount of miR-126-3p detected in the plasma of each HCC patient during sorafenib treatment. Each line represents one patient, and the dots indicate the levels of miR-126-3p at each blood withdrawal time point (one per month, Tn) normalized on the miR-126-3p level before the start of treatment (T0). (**B**) Focus on miR-126-3p early levels after sorafenib administration. T_n_ indicates the sequential blood withdrawal time point carried out once *per* month. One-way ANOVA, followed by Tukey’s test, was used to compare miR-126-3p levels among three time points (T0, T1 and T2).

We previously reported that the levels of GAS5 and miR-126-3p were respectively lower and higher in plasma of untreated HCC patients compared to healthy individuals [20]. Here, we measured the levels of these ncRNAs in the plasma of HCC patients during the treatment with sorafenib. In particular, we analyzed the longitudinal levels of GAS5 and miR-126-3p in plasma of 7 HCC patients treated with sorafenib for several months (3–12) by qPCR. Plasma was collected before the beginning of the treatment (T_0_) and once *per* month during therapy (T_n_) as described in Section 2. As shown in Figure 1A and Figure 2A, we observed different levels of GAS5 and miR-126-3p during the follow-up of the patients and at relatively long periods of time (i.e., case 11-LB-01). We focused our attention on the early time points (T_0_, T_1_ = one month after treatment started, T_2_ = two months after treatment started) since they were available for all patients. As shown in Figure 1B, four out of seven patients (38-LB-01, 55-LB-01, 61-LB-01, 92-LB-01) showed an increase in the level of plasma GAS5 after the first month of treatment with sorafenib (T_1_) when compared to the basal condition (T_0_). On the contrary (Figure 2B), four of seven patients (11-LB-01, 73-LB-01, 92-LB-01 and 136-LB-01) showed a decrease in the amount of miR-126-3p in the plasma after the first month of treatment with sorafenib (T_1_) when compared to the basal condition (T_0_).

**Figure 3:**
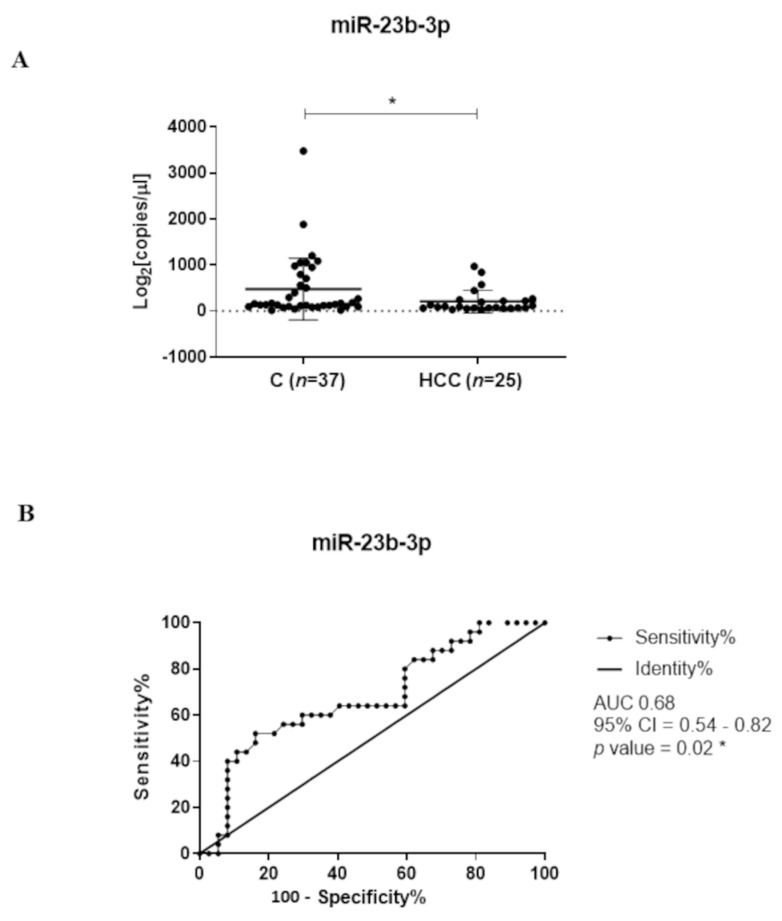
ddPCR determination of plasma levels of miR-23b-3p in HCC patients. (**A**) The graph shows the amount of miR-23b-3p detected in the plasma of healthy individuals (C) and HCC patients. The Mann–Whitney U-test was used to compare the two groups; * indicates a *p*-value < 0.05. (**B**) ROC curve analysis of miR-23b-3p in HCC patients and healthy individuals. AUC = Area Under the Curve; CI = Confidence Interval.

As shown in Figure 3, we also focused on circulating miR-23b-3p because we previously shown that miR-23b-3p was down-modulated in HCC tissues compared to peritumoral tissues, and its expression could contribute to the molecular characterization of HCC [23]. Here, we first evaluated the plasma levels of miR-23b-3p in 25 untreated HCC patients and 37 healthy control individuals, as we had already determined the circulating levels of GAS5 and miR-126-3p in the same cohorts [20]. For miR-23b-3p, we used ddPCR since we verified that high Ct values were observed when qPCR was used (data not shown), reaching the limit of detection of the technique.

We found a significant lower level of circulating miR-23b-3p in HCC patients compared to healthy individuals (680.4 ± 161.1 copies/µL and 1580 ± 367.1 copies/µL, respectively; *p*-value= 0.018; Figure 3A). As shown in Figure 3B, ROC curve analysis revealed the potential diagnostic performance of miR-23b-3p in discriminating HCC from healthy subjects (AUC = 0.68; *p*-value = 0.02).

Finally, the plasmatic levels of miR-23b-3p resulted as significantly associated with the tumor grading (*p*-value = 0.041). No statistically significant correlations were found between miR-23b-3p levels and gender, age, the presence of hepatitis and liver cirrhosis (Table 1).

**Figure 4:**
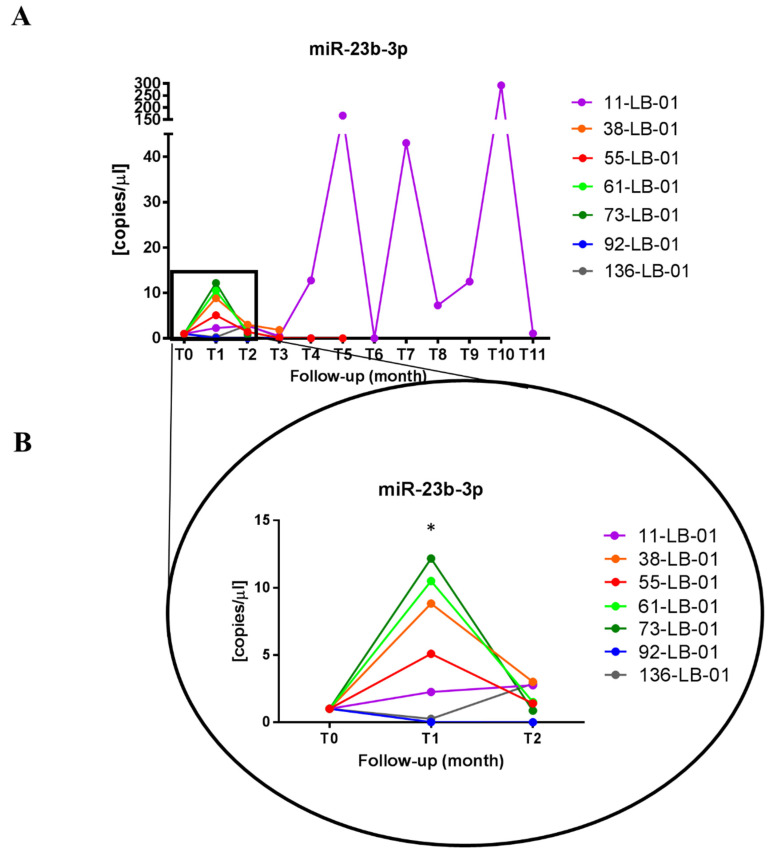
miR-23b-3p levels in the plasma of HCC patients treated with sorafenib. (**A**) The graph shows the amount of miR-23b-3p detected in the plasma of each HCC patient during sorafenib treatment. Each line represents one patient, and the dots indicate the levels of miR-23b-3p at each blood withdrawal time point (one per month, Tn) normalized on the miR-23b-3p level before the start of treatment (T0). (**B**) Focus on miR-23b-3p early levels after sorafenib administration. T_n_ indicates the sequential blood withdrawal point carried out once per month. One-way ANOVA, followed by Tukey’s test, was used to compare miR-23b-3p levels among three time points (T0, T1, and T2), * indicates a *p*-value < 0.05 versus T0.

Following these findings, in order to verify whether sorafenib may influence the circulating levels of miR-23b-3p, we measured its amount in the plasma of treated HCC patients. As shown in Figure 4A, we found that during the follow-up, patients displayed different levels of miR-23b-3p. The average amount of miR-23b-3p significantly increased one month after starting sorafenib treatment (T_1_) with respect to T_0_ (*p*-value = 0.024) (Figure 4B). We observed a distinct increase in the amount of miR-23b-3p detected in five out of seven patients (11-LB-01, 38-LB-01, 55-LB-01, 61-LB-01 and 73-LB-01), then at T_2_, miR-23b-3p levels decreased.

## 4. Discussion

Continuous efforts are performed to discover eligible biomarkers for early detection, monitoring, prognosis and prediction of treatment response in cancer patients, including hepatocellular carcinoma. The aberrant expression of ncRNAs in HCC tissues, their extracellular release and stability led to probe their use as circulating diagnostic and prognostic tools for HCC [32]. Moreover, in the era of personalized medicine, the information obtained from the blood of patients may be paramount in assessing the disease outcome [33]. Serum α-fetoprotein (AFP), des-gamma carboxyprothrombin (DCP), and Dickkopf-1 (DKK1) proteins have already been described as circulating biomarkers of HCC. However, the use of AFP and DKK1 lack in sensitivity and reliability, while DCP amount reproducibility is controversial [34,35]. In order to meet the urgent need in the identification of predictive molecules to indicate responsiveness or resistance to sorafenib therapy, we set up a longitudinal study to analyze the circulating levels of different ncRNAs in patients treated with sorafenib.

By using a disease/pathway-array of lncRNAs and miRNAs in the HA22T/VGH in vitro model of HCC, we previously reported that GAS5 and miR-126-3p resulted in up- and down-regulation, respectively, in a dose-dependent manner upon sorafenib treatment. We also found that their levels in plasma acted as potential diagnostic biomarkers in distinguishing HCC patients from healthy subjects [20]. In this exploratory work, we aimed to obtain further insights by analyzing the longitudinal circulating levels of GAS5 and miR-126-3p levels in the plasma of HCC patients during several months of treatment with sorafenib.

Fayda et al. described a significant increase in plasma GAS5 levels in head and neck cancer patients undergoing current chemotherapy treatments when patients with partial response/progressive disease were compared to patients with complete response [36]. In malignant pleural mesothelioma patients, the plasmatic circulating levels of GAS5 resulted decreased after chemotherapy [37]. The only data available regarding changes of miR-126-3p in the plasma in association with therapy derives from uveal melanoma (UM) and metastatic colorectal cancer (mCRC). Triozzi et al. described a decrease in miR-126-3p levels in UM patients treated with adjuvant dacarbazine and interferon-α-2b, but no association with outcome was observed [38]. Hansen et al. described an increase in miR-126-3p levels in non-responder patients compared to responders exploring the use of plasma levels of miR-126-3p as outcome predictor value in mCRC during first-line chemotherapy combined with bevacizumab [39]. To our knowledge, for the first time in the current longitudinal study, we examined the levels of GAS5 and miR-126-3p in plasma of HCC patients treated with sorafenib. Particularly, we observed distinct level variations among each patient during the whole follow-up but also a common trend of variation of a specific ncRNA in the different patients treated with sorafenib when we focused the attention on the T_1_. The absence of constant levels, but conversely a general fluctuation during the follow-up, may suggest their potential sensitivity to the drug and/or specific clinical conditions of the patients acquired during treatment. Therefore, we hypothesize considering the possible role of GAS5 and miR-126-3p in response to sorafenib in HCC in a future study that enrolls a larger cohort of HCC patients treated with sorafenib.

Concerning miR-23b-3p, in this study, we first assessed whether plasma miR-23b-3p levels might be different between healthy subjects and untreated HCC patients. The significant downregulation of circulating miR-23b-3p levels in untreated HCC patients compared to healthy individuals and the results of ROC analysis suggest that miR-23b-3p may have a potential clinical value. To our knowledge, only two other studies explored the amount of miR-23b-3p in plasma of cancer patients using qPCR. Kou et al. demonstrated that plasma miR-23b-3p levels were significantly downregulated in colorectal cancer (CRC) patients compared to healthy individuals. Interestingly, CRC patients with low miR-23b-3p levels in plasma exhibited a worse prognosis than those with high levels [40]. On the contrary, Kun et al. demonstrated that miR-23b-3p levels in the plasma of gastric cancer (GC) patients were significantly higher when compared to healthy individuals and that GC patients with elevated miR-23b-3p levels showed a lower either disease-free or overall survival than those with low plasmatic levels [41]. These discrepancies may be due to the intrinsic dual biological role of miR-23b-3p as tumor-suppressor/onco-miR [21], the different cancer types and the altered molecular mechanisms involved. Our study is the first one highlighting the differences in plasma levels of miR-23b-3p between untreated HCC patients and healthy individuals.

The evaluation of the amount of miR-23b-3p in the plasma of patients treated with sorafenib during the follow-up displayed a significant increase in secreted miR-23b-3p one month after starting the treatment, followed by a drop. Considering its biological role as a tumor suppressor in HCC, it is interesting that the administration of the multikinase inhibitor would boost its levels. The systemic effect of the drug may also influence miR-23b-3p release from cells, others from the tumor mass, thus contributing to an increase in miR-23b-3p levels in the bloodstream. Taken together, these findings suggest that miR-23b-3p may have a diagnostic value for HCC, and it might also be not excluded as a circulating molecule sensitive to sorafenib treatment to be confirmed in a wide cohort.

In summary, to the best of our knowledge, this study is the first one highlighting the diagnostic value of plasma miR-23b-3p in untreated HCC and the determination of circulating GAS5, miR-126-3p and miR-23b-3p in HCC patients treated with sorafenib. The limited number of patients in follow-up recruited is due to the difficulties in obtaining sequential liquid biopsies from HCC patients treated with sorafenib because they are rarely collected. Probably for this reason, at present, no correlation has been identified between the circulating levels of the studied ncRNAs and the clinicopathological features or clinical outcomes of patients treated with sorafenib. From a general point of view, our data constitute a proof of concept of the need and usefulness of collecting liquid biopsy samples at different time points during the follow-up of HCC patients treated with TKI. The future directions to this study will be the determination of circulating miR23b-3p levels in larger cohorts of healthy controls and untreated HCC patients to confirm the diagnostic value of plasma miR-23b-3p. In addition, miR-23b-3p will be examined in a cohort of cirrhotic patients who have not yet developed HCC as a possible biomarker for an early diagnosis of HCC. Regarding the determination of ncRNAs levels in response to the treatment of HCC patients with sorafenib, or other TKI, the future direction to advance in this field of biomedicine will be to pursue a strong pragmatic and scientific collaboration between basic scientists and oncologists in various hospitals; this will aid in organizing effective recruitment of longitudinal liquid biopsy specimens of HCC patients during TKI treatment.

## Figures and Tables

**Table 1:** Associations between the clinicopathological characteristics of HCC patients and the plasmatic levels of miR-23b-3p ^$^.

KERRYPNX	Number of Patients	Low Copies/µL ≤ 305	High Copies/µL > 305	*p*-Value
**Gender**				0.604
Male	21	11	10	
Female	4	3	1	
**Age ^#^**				0.695
≤73 years	13	6	7	
>73 years	12	7	5	
**Grading**				0.041
G1–G2	14	5	10	
G3	10	8	2	
**Hepatitis B infection**				0.593
Yes	4	3	1	
No	21	10	11	
**Hepatitis C infection**				0.695
Yes	12	7	5	
No	13	6	7	
**Cirrhosis**				0.238
Yes	12	8	4	
No	11	5	8	
**Recurrence after surgery**				1.000
Yes	17	9	8	
No	8	4	4	
**Tumor Size**				0.642
>5 cm	6	4	2	
<5 cm	14	7	7	

**^$^** The median value of plasmatic copies/µL was chosen as cut-off point. **^#^** The median value of years was used as the cut-off point.

## Supplementary Materials

The following are available online at https://www.mdpi.com/article/10.3390/biomedicines9070813/s1, Table S1: Characteristics of the 7 HCC patients treated with sorafenib enrolled in the longitudinal study; Table S2: Clinical characteristics of HCC patients enrolled in the study.
