# Supplementary material for: Longitudinal Circulating Levels of miR-23b-3p, miR-126-3p and lncRNA GAS5 in HCC Patients Treated with Sorafenib
Source: Biomedicines. 2021 Jul 13;9(7):813. doi: 10.3390/biomedicines9070813 (PMC8301380; doi:10.3390/biomedicines9070813)
Supplement: Supplementary file 1 [file biomedicines-09-00813-s001.zip › Table S2.pdf]

Table S2. Clinical characteristics of HCC patients enrolled in the study.

CASE	GENDER	AGE (y)	GRADING	BACKGROUND DISEASE	HBV	HCV	AFP (ng/mL)
342	M	75	G2-G3	STEATOSIS	-	+	4
343	M	64	G2	CHRONIC HEPATITIS	-	+	1
345	M	72	G2	CHRONIC HEPATITIS	–	+	–
347	M	63	G2	ACTIVE CIRRHOSIS	–	–	8
348	M	60	G3	CHRONIC HEPATITIS	+	–	10
349	M	71	G2	HEPATITIS	–	–	-
350	M	78	G3	CHRONIC HEPATITIS	–	+	3
351	F	69	G2	ACTIVE CIRRHOSIS	–	+	–
352	M	82	G2	EARLY CIRRHOSIS	–	+	–
353	M	82	G2	NORMAL	–	–	763
354	M	71	G1	STEATOSIS	–	–	–
355	F	78	G2	STEATOSIS	–	–	–
357	M	57	G3	CIRRHOSIS WITH STEATOSIS	+	–	17
359	M	75	G2	CIRRHOSIS	–	+	–
360	M	71	G1	ACTIVE CIRRHOSIS	–	–	–
361	M	74	G2	EARLY CIRRHOSIS WITH MODERATE STEATOSIS	–	+	–
362	F	79	G2–G3	CIRRHOSIS WITH STEATOSIS	–	+	13
363	M	74	G2	CIRRHOSIS WITH STEATOSIS	–	–	–
364	F	77	G3	CIRRHOSIS WITH STEATOSIS	–	+	7
365	M	76	G3	VON MEYENBURG COMPLEX	–	–	15
366	M	65	G2–G3	CIRRHOSIS WITH STEATOSIS	+	–	–
367	M	73	G2	CHRONIC HEPATITIS	–	+	9
368	M	73	G3	CHRONIC HEPATITIS	+	–	7
369	M	68	G3	CIRRHOSIS WITH STEATOSIS	–	–	–
370	M	74	G2	CHRONIC HEPATITIS	–	+	–
