# Supplementary material for: Longitudinal Circulating Levels of miR-23b-3p, miR-126-3p and lncRNA GAS5 in HCC Patients Treated with Sorafenib
Source: Biomedicines. 2021 Jul 13;9(7):813. doi: 10.3390/biomedicines9070813 (PMC8301380; doi:10.3390/biomedicines9070813)
Supplement: Supplementary file 1 [file biomedicines-09-00813-s001.zip › Table S1.pdf]

Table S1. Characteristics of the 7 HCC patients treated with sorafenib enrolled in the longitudinal study.

	11-LB-01	38-LB-01	55-LB-01	61-LB-01	73-LB-01	92-LB-01	136-LB-01
Sex	M	M	F	M	M	M	F
Age (y)	75	63	50	32	83	72	73
Background disease	Cirrhosis and steatosis	Cirrhosis and viral hepatitis	Cirrhosis and viral hepatitis	Cirrhosis and viral hepatitis	Normal liver	Cirrhosis	Cirrhosis
HBV	-	-	+	+	-	-	-
HCV	-	+	-	-	-	-	-
Grading	2	N/A	N/A	3	N/A	N/A	N/A
AFP (ng/ml)	102.8	76.4	2.4	106,408.7	353.5	104.9	74.1
Local treatments	HCC surgical resection	No	No	No	No	TACE	No
Tumor spread at diagnosis	Liver	Liver and abdominal lymphonodes	Liver	Lungs metastasis	Liver	Liver	Liver, bones and lungs metastasis
N° of blood sample collected (months)	12	4	6	3	3	6	4
PFS (months)	11	7	5	2	3	5	2
OS (months)	42	25	19	5	7	20	ALIVE

M: Male; F: Female; y: years; N°: number; HBV: Hepatitis B Virus; HCV: Hepatitis C Virus; AFP: α-fetoprotein; N/A: not applicable; TACE: Transarterial Chemoembolization; PFS: Progression-Free Survival; OS: Overall survival.
